# Predictive Analytic Model for Diagnosis of Ectopic Pregnancy

**DOI:** 10.3389/fmed.2021.646258

**Published:** 2021-04-29

**Authors:** Ploywarong Rueangket, Kristsanamon Rittiluechai

**Affiliations:** Department of Obstetrics and Gynecology, Phramongkutklao Hospital, Bangkok, Thailand

**Keywords:** ectopic pregnancy, first trimester complications, predictive scoring model, pregnancy of unknown location, serum human chorionic gonadotropin, statistical model

## Abstract

**Objective:** Ectopic pregnancy (EP) is a serious condition. Delayed diagnosis could lead to life-threatening outcomes. The study aimed to develop a diagnostic predictive model for EP to approach suspected cases with prompt intervention before the rupture occurred.

**Methods:** A retrospective cross-sectional study enrolled 347 pregnant women presenting first-trimester complications (abdominal pain or vaginal bleeding) with diagnosis suspected of pregnancy of unknown location, who were eligible and underwent chart review. The data including clinical risk factors, signs and symptoms, serum human chorionic gonadotropin (hCG), and ultrasound findings were analyzed. The statistical predictive score was developed by performing logistic regression analysis. The testing data of 30 patients were performed to test the validation of predictive scoring.

**Results:** From a total of 22 factors, logistic regression method–derived scoring model was based on five potent factors (history of pelvic inflammatory disease, current use of emergency pills, cervical motion tenderness, serum hCG ≥1,000 mIU/ml, and ultrasound finding of adnexal mass) using a cutoff score ≥3. This predictive index score was able to determine ectopic pregnancy with an accuracy of 77.8% [95% confidence interval (CI) = 73.1–82.1], specificity of 91.0% (95% CI = 62.1–72.0), sensitivity of 67.0% (95% CI = 88.0–94.0), and area under the curve of 0.906 (95% CI = 0.875–0.937). In the validation group, no patient with negative result of this score had an EP.

**Conclusion:** Statistical predictive score was derived with high accuracy and applicable performance for EP diagnosis. This score could be used to support clinical decision making in routine practice for management of EP.

## Introduction

Ectopic pregnancy (EP) is a potentially life-threatening complication when treatment is delayed. Reported maternal death by Confidential Enquiry into maternal deaths (CEMD) in the United Kingdom researcher as the fourth leading cause of death (1). Despite a comprehensive medical evaluation including patient risk assessment, clinical evaluation, and investigations, the rate of deaths in the United Kingdom has not declined since 1991. In fact, one-third of women with EP have no clinical signs, and up to 10% are asymptomatic (2, 3). The use of transvaginal ultrasonography (TVS) was found to improve the accuracy of diagnosis. However, only 73.9% of tubal EPs were visualized by initial TVS (4).

Until recently, serial measurements of serum human chorionic gonadotropin (hCG) levels had been shown to benefit EP detection. Although with many research data, the serum hCG's patterns were still unable to differentiate those with EP from intrauterine pregnancy or abortion precisely enough (5). Even though the longer follow-up serum hCG may help to locate gestation, 32% of ruptured EP usually would occur within the first 48 h, which is the most critical timing for physicians to offer the intervention before the rupture. Unfortunately, the uncertainty remained in the initial diagnosis leading to high mortality and infertility risk for the next pregnancy (6).

Attempts to develop a predictive model began in the early 20th century for early diagnosis patients with EP by using clinical data, serum marker (hCG and/or progesterone), and ultrasound finding. One widely known statistical model using serum hCG to predict EP is the M1 and M4 model by Condous et al. (7). Whereas, the first M1 model seems to have good sensitivity and specificity, the positive predictive value is still low. Later, the M4 model, based on serial serum hCG values 48 h apart, demonstrated a better predictive ability but lower performance in different populations (8).

The challenge in identifying the EP among patients presenting early pregnancy complications is crucial, because early detection could reduce maternal morbidity and mortality and also preserved future fertility. Despite much data and research, no infallible EP predictive model is available. This research aimed to create an effective predictive model to diagnose EP and extract the association factors inside the data.

## Materials and Methods

### Study Designs and Subjects

The cross-sectional study was reviewed and approved by the Royal Thai Army Medical Department Institutional Review Board before conducting the study using electronic medical records of Phramongkutklao Hospital between October 2010 and April 2020, involving women presenting vaginal bleeding and/or abdominal pain during the first trimester of pregnancy. All patients underwent transvaginal ultrasound. Inclusion criteria comprised patients who had been evaluated and diagnosed with pregnancy of unknown location (PUL), pregnancy with no signs of intrauterine pregnancy, or an extrauterine pregnancy *via* ultrasonography at the first visit. Patients presenting signs clinically suggestive of ruptured EP (clinical instability or sign of intra- abdominal hemorrhage) or who show any evidence of intrauterine gestational content or EP (adnexal mass consisted of fetal pole or fetal heart motion) by ultrasound at the first visit were excluded.

The primary outcome of interest was the final diagnosis of EP or non-EP. The diagnosis of EP was confirmed by postoperative pathological result and abnormality in pattern measurement of serial hCG levels (plateau or abnormal increased/decreased patterns) with unidentified chorionic villi in uterine cavity or having undergone treatment with medication. In the non-EP group, final diagnosis included threatened abortion, spontaneous abortion, or miscarriage. They needed to be confirmed by pathological result or subsequent decrease of serum hCG level in miscarriage pattern or continuing intrauterine pregnancy by subsequent ultrasound finding.

Demographic data, history of risk factors, clinical manifestations, initial serum hCG levels, and ultrasound results were reviewed and recorded, totaling 22 factors. All factors were extracted and selected from literature reviews to obtain statistical significance relevant to our research outcome.

### Statistical Analysis

The method used for EP prediction was logistic regression. Collected data were checked, coded, and then analyzed using STATA 15.1/IC (StataCorp). The data were chronologically split up in a training and a test set of 9:1 ratio. In a training dataset, frequency distribution of demographic characteristics and factors were calculated to determine descriptive statistics of the samples. Binary logistic regression analysis was used to determine the risk factors associated with EP. The magnitude of association was presented as crude odds ratios (ORs) with 95% confidence interval (CI). A *p* < 0.05 was considered as statistically significant. Differences identified between subgroups of patients are considered hypothesis-generating and require confirmation in independent studies. Multivariate analysis was performed to adjust confounders by using logistic regression analysis with enter method, which is a default function of STATA Software to simultaneously eliminate (backward stepwise elimination) independent factors in the model. All statistically significant predictive factors in multivariate analysis were used to create score. The probability of EP diagnosed with each score value was calculated, and then sensitivity, specificity, and accuracy of various score cutoff were computed. Maximum likelihood estimation was used to obtain coefficients from logistic regression analysis. The score was applied to the testing dataset for validation. Regression coefficient- based scoring algorithms were performed, and scoring system for EP diagnostic model was derived.

## Results

### Characteristics of Study Populations

From a total of 1,275 pregnant women presenting at early pregnancy with complications, 347 (27%) pregnant women with suspected PUL at initial diagnosis were identified. The mean age was 30 years with 43.8% nullipara. In 347 patients with PUL, 55% (*n* = 191) were EP, and 45% (*n* = 156) were non-EP. Among 156 patients with non-EP, 20 (12.8%) were threatened abortion, one (0.6%) blighted ovum, one (0.6%) corpus luteal leakage, and other 134 (85.9%) were spontaneous abortion. The majority of patients with EP had no underlying disease (88.5%), no history of pelvic surgery (79.6%), no previous EP (95.3%), and no history of pelvic inflammatory disease (PID) (90.6%). There was no statistically significant difference in gestational age (GA) at diagnosis between EP patients (52.6 ± 16.1 days) and non-EP patients (50.7 ± 15.8 days). Ninety-seven percent of patients with EP were non-smoker, and only 16.6% were in current use of emergency pill. Regarding clinical presentation and assessment, 87.4% of patients in the EP group had abdominal pain, and 74.3% of them had noticed abnormal vaginal bleeding. There was abdominal tenderness in 69.1% of patients with EP, and 37.7% had cervical motion tenderness. On the other side, 35.6% (68 of 191) of patients with EP presented with serum hCG ≥1,000, whereas 45% of patients with EP did not obtain blood examination. When compared with patients with no EP, complex adnexal mass and free fluid in cul-de-sac by initial ultrasound finding were more common in EP patients, which was 87.4 vs. 17.3% and 62.3 vs. 17.3%, respectively, as presented in Table 1.

**Table 1:** Demographic characteristics of the study population.

	**Characteristics**	**Total (*N* = 347)** ***N* (%)**	**EP (*N* = 191)** ***N* (%)**	**Non-EP (*N* = 156)** ***N* (%)**	***P*-value**
Risk factor	Age (years) mean ± SD	30.1 ± 6.2			
	Age group (years)				0.497^a^
	<35	264 (76.1)	148 (56.1)	116 (43.9)	
	≥35	83 (23.9)	43 (51.8)	40 (48.2)	
	BMI (kg/m^2^)				0.963^a^
	<23	234 (67.4)	129 (55.1)	105 (44.9)	
	≥23	113 (32.6)	62 (54.9)	51 (45.1)	
	Parity				0.218^a^
	Nulliparity	152 (43.8)	78 (51.3)	74 (48.7)	
	Multiparity	195 (56.2)	113 (57.9)	82 (42.1)	
	Gestational age at diagnosis (days)		52.6 ± 16.1	50.7 ± 15.8	0.131^b^
	Underlying disease				0.292^a^
	No	301 (86.7)	169 (56.1)	132 (43.9)	
	Yes	46 (13.3)	22 (47.8)	24 (52.2)	
	History of pelvic surgery				0.356^a^
	No	261 (78.6)	152 (58.2)	109 (41.8)	
	Yes	71 (21.4)	37 (52.1)	34 (47.9)	
	Smoking				0.051^a^
	No	327 (98.5)	185 (56.6)	142 (43.4)	
	Yes	5 (1.5)	5 (100.0)	0	
	History of ectopic pregnancy				0.880^a^
	No	329 (95.6)	182 (55.3)	147 (44.7)	
	Yes	15 (4.3)	8 (53.3)	7 (46.7)	
	History of PID				0.089^a^
	No	291 (95.7)	173 (59.4)	118 (40.6)	
	Yes	13 (4.3)	11 (84.6)	2 (15.4)	
	Current use of emergency pill				0.001^a^
	No	285 (88.8)	156 (54.7)	129 (45.3)	
	Yes	36 (11.2)	31 (86.1)	5 (13.9)	
	Assisted reproductive technology				0.954^a^
	No	332 (96.8)	184 (55.4)	148 (44.6)	
	Yes	11 (3.2)	6 (54.6)	5 (45.4)	
Symptoms	Abdominal pain				<0.001^a^
	No	67 (19.4)	23 (34.3)	44 (65.7)	
	Yes	279 (80.6)	167 (59.9)	112 (40.1)	
	Abnormal vaginal bleeding				0.087^a^
	No	77 (22.2)	49 (63.6)	28 (36.4)	
	Yes	270 (77.8)	142 (52.6)	128 (47.4)	
	Nausea, vomiting				0.503^a^
	No	219 (84.6)	130 (59.4)	89 (40.6)	
	Yes	40 (15.4)	26 (65.00)	14 (35.00)	
	Faint				0.016^a^
	No	241 (91.6)	139 (57.7)	102 (42.3)	
	Yes	22 (8.4)	19 (86.4)	3 (13.6)	
Signs	Abdominal tenderness				<0.001^a^
	No	172 (49.6)	59 (34.3)	113 (65.7)	
	Yes	175 (50.4)	132 (75.4)	43 (24.5)	
	Cervical motion tenderness				<0.001^a^
	No	271 (78.8)	116 (42.8)	155 (57.2)	
	Yes	73 (21.2)	72 (98.6)	1 (1.4)	
Serum marker	Serum hCG level at first visit (mIU/ml)				<0.001^a^
	<1,000	127 (53.1)	37 (29.1)	90 (70.9)	
	≥1,000	112 (46.9)	68 (60.7)	44 (39.3)	
Ultrasound findings	Intra-uterine anechoic content				0.908^a^
	No	308 (89.5)	168 (54.6)	140 (45.4)	
	Yes	36 (10.5)	20 (55.6)	16 (44.4)	
	Endometrial thickness >14 mm				0.461^a^
	No	273 (83.8)	149 (54.6)	124 (45.4)	
	Yes	53 (16.2)	26 (49.1)	27 (50.9)	
	Adnexal mass of complex echogenicity				<0.001^a^
	No	152 (43.4)	23 (15.1)	129 (84.9)	
	Yes	194 (56.1)	167 (86.1)	27 (13.9)	
	Free fluid in cul-de-sac				<0.001^a^
	No	195 (57.2)	67 (34.4)	128 (65.6)	
	Yes	146 (42.8)	119 (81.5)	27 (18.5)	

*SD, standard deviation; BMI, body mass index; PID, pelvic inflammatory disease*.

a *χ^2^ Test*.

*bt Test*.

Using logistic regression in the training dataset, from a total of 22 factors, the results highlighted eight significant factors in univariate analysis, exhibiting an established relationship with EP, including current use of emergency pill, abdominal pain, fainting, abdominal tenderness, cervical motion tenderness, initial serum hCG level ≥1,000 mIU/ml, ultrasound findings of complex echogenicity, and finding of free fluid in cul-de-sac. Although history of PID and previous EP were not found to be statistically significant factors in our univariate analysis, these factors were proven to be clinically significant as risk factors of EP from many literature reviews (9–12). Thus, we selected history of PID and previous EP to evaluate in multivariate analysis. Stepwise method using backward elimination for factor selection was used in multivariate analysis, based on predictors and significant *p* < 0.2. We found that the variables history of PID, current use of emergency pill, cervical motion tenderness, initial serum hCG level ≥1,000 mIU/ml, and ultrasound findings of complex echogenicity were statistically significant factors to predict EP, as shown in Table 2.

**Table 2:** Univariate and multivariate logistic regression analyses of the factors significantly associated with ectopic pregnancy and scoring system.

**Features** ** total case (*N*) = 347**	**Missing data** ** (%)**	**Univariate analysis**		**Multiple analysis**			**Risk score (13)**
		**Crude ORs** ** (95% CI)**	***P*-value**	**Adjusted ORs** ** (95% CI)**	***P*-value**	**Coefficient**	
History of PID	43 (12.3%)	3.75 (0.82–17.23)	0.089	7.30 (0.63–85.08)	0.112	1.99	2
History of ectopic pregnancy	3 (0.86%)	0.92 (0.33–2.60)	0.880				
Emergency pill	26 (7.5%)	5.13 (1.94–13.56)	0.001	4.51 (0.92–22.05)	0.063	1.51	1.5
Abdominal pain	1 (0.3%)	2.85 (1.63–4.98)	<0.001				
Faint	84 (24.2%)	4.65 (1.34–16.13)	0.016				
Abdominal tenderness	—	5.88 (3.69–9.37)	<0.001				
Cervical motion tenderness	3 (0.8%)	96.21 (13.18–702.50)	<0.001	9.03 (4.36–18.70)	<0.001	2.20	2.5
Serum hCG level at first visit(mIU/ml) ≥1,000	108 (31.1%)	3.76 (2.19–6.44)	<0.001	2.63 (1.27–5.46)	0.009	0.96	1
Adnexal mass of complex echogenicity	1 (0.3%)	34.69 (19.01–63.32)	<0.001	9.83 (1.20–80.73)	0.033	2.29	2.5
Free fluid in cul-de-sac	6 (1.7%)	8.42 (5.05–14.05)	<0.001				

*ORs, odds ratios; CI, confidence interval; PID, pelvic inflammatory disease*.

The score of each five significantly predictive factors was adjusted according to the coefficient-based scoring ratio in multiple logistic regression, as revealed in Table 2. The statistical model was developed as a predictive scoring model with the acronym APrIlE score, as presented in Table 3. Thus, a predictive index score with cutoff level ≥3 provided prediction of patients with EP with the best specificity of 91.0% (95% CI = 62.1–72.0), with a good balance in sensitivity of 67% (95% CI = 88.0–94.0). The area under the curve (AUC) was 0.906 (95% CI = 0.875–0.937), as presented in Table 4. Lastly, the model was validated in a new group of patients with PUL (*n* = 30). We found that the predictive index score with cutoff ≥3 could provide an exceptional performance of 90% (95% CI = 73.5–97.9) in accuracy, specificity of 100% (95% CI = 83.9–100), and sensitivity of 66.7% (95% CI = 29.9–92.5), with AUC of 0.905 (95% CI = 0.875–0.937). Among patients who were classified by the score in the low-risk group of EPs, none had been diagnosed with EP.

**Table 3:** Predictive ectopic pregnancy scoring model, with the acronym APrIlE.

**Risk factor**	**Adnexal mass of complex echogenicity**	**Pain related to cervix**	**Infection of pelvis**	**≥1,000 of serum hCG**	**Emergency pill current use**
Score	2.5	2.5	2	1	1.5

**Table 4:** Predictive scoring system cutoff detailed report performance.

**Detailed report of sensitivity and specificity**					
**Cutoff**	**Sensitivity**	**Specificity**	**Corrected classified**	**LR+**	**LR–**
≥1	94.8%	58.3%	78.4%	2.27	0.09
≥1.5	91.1%	79.5%	85.9%	4.44	0.11
≥2	90.1%	81.4%	86.2%	4.84	0.12
≥2.5	89.5%	82.1%	86.2%	4.99	0.13
≥3	67.0%	91.0%	77.8%	7.47	0.36
≥3.5	65.1%	91.0%	77.2%	7.35	0.37
≥4	46.1%	98.1%	69.5%	23.96	0.55
≥4.5	41.4%	99.4%	67.4%	64.52	0.59

*LR+, positive likelihood ratio; LR–, negative likelihood ratio*.

## Discussion

Overall, the data of 347 patients were analyzed by using univariate analysis, and 22 factors were studied. From a total 1,275 pregnant patients presenting first-trimester complications, 27.2% were diagnosed with PUL. The incidence was similar to many studies, ranging between 7 and 31% (14–17). In our study, EP was diagnosed in 55% of PUL, which was similar to the 43% rate in one large prospective observational published study by Malek-Mellouli et al. (18). However, another retrospective study reported the wide range of 7 to 20% (15–19), although a limitation of retrospective studies was that the complete definition to diagnose spontaneous resolution of EP or non-EP in PUL might be failed, as the true location of gestation was never known. In our study, we used the pathological report with unidentified chorionic villi in uterine cavity and the normal decline in serum hCG patterns to confirm miscarriage diagnosis.

The age of 35 years appears to be a risk factor of EP stated by the American College of Obstetricians and Gynecologists (ACOG) in 2018 (20). However, our study found that there was no statistically significant difference in EP and non-EP between the age groups <35 and ≥35 years. This finding was similar to the multicenter case-control study by Cheng Li et al., who reported that age group was not a significant risk factor for EP (12). On the other hand, it appears that the mean age of our study population was 30.1 ± 6.2 years. The study by Essa et al. (21), also revealed that the majority of patients were in their 30s. Also, these findings match those of another large case-control study by Bouyer et al. (22), which found that 51.6% of patients was 30 years old. So far, we only believe that the age at 30s could be a piece of a jigsaw puzzle that needed the matching pieces before the EP puzzle could be solved. As in the complex data to predict a single disease, only one factor could not be used to solve the problem. Another interesting finding was the GA at diagnosis. The mean GA at diagnosis of EP patients was 52.6 ± 16.1 days (7.5 ± 2.3 weeks) and 50.7 ± 15.8 days (7.2 ± 2.2 weeks) in non-EP patients. These results share similarities with a study by Saxon et al., in which the mean GAs at diagnosis of an unruptured tube and of those with a ruptured tube were 6.9 ± 1.9 and 7.2 ± 2.2 weeks, respectively (23). Despite that our study could not identify the differences in GA at diagnosis between the EP and non-EP groups, the systematic review study revealed that the first presenting symptoms of miscarriage most likely occurred around 5 weeks and 8 to 10 weeks of GA (24).

Theoretically, any condition that delays or interferes with the passage of an embryo through the fallopian tubes may increase the risk of EP (25). Despite the study by Berek et al., (26), which explained that up to one-half of EP cases have unidentified risk factors, a history of PID has a strong association with EP in our study. Also, from many studies (9–12), PID was also found to be one of the traditional risk factors and could be explained by its pathophysiology causing inflammation and disruption of tubal motility. Emergency contraceptive pills are another factor that could impair motility of fallopian tube. However, the use of emergency pills related to EP is still inconclusive from various studies (12, 27–29). In the result, using a different population, the treatment failure of emergency contraceptive pills was found to be related to EP. According to a history of previous EP in this study, there were only 4% of the patients in each study group who had a history of previous EP and shown insignificance relevant to EP. Although a case-control study reported that a history of previous EP was the strongest risk factor associated with EP (OR = 17.16, 95% CI = 1.89–155.67) (29), other studies could not demonstrate the association between previous EP and subsequent EP (18, 21).

Several studies have found the association between cigarette smoking and EP (22, 29, 30). Despite the fact that pathophysiology of smoking related to EP remains unclear, inhalation of cigarette smoking has shown the effect on the function of cilia and smooth muscles of fallopian tube presented by animal models (25). In this study, we found that only five patients were smokers, and all of them were diagnosed with EP. However, there was no significant difference between the two groups.

Many studies attempted to address clinical symptoms associated with EP. As noted by Malek-Mellouli et al. (18), abdominal pain with vaginal bleeding during the first trimester was strongly associated with EP. In addition to the study of Buckley et al. (31), the developed clinical prediction model for EP showed that cervical motion and abdominal tenderness were addressed as high and moderate risk factors for EP, respectively. Similarly, we found that abdominal pain, vaginal bleeding, cervical motion, and abdominal tenderness were shown to be the potent risk factors. However, we acknowledged that fainting was more relevant to EP than abdominal tenderness (86 vs. 60%) in comparison with non-EP. However, 10 to 30% of patients with EP usually presented with unspecific clinical signs and symptoms (2, 26).

In patients with inconclusive symptom, ultrasound findings became an important investigation for diagnostic EP. Inhomogeneous adnexal mass separated from ovary and free fluid in cul-de-sac are important findings that could be related to EP. These two factors have sensitivity of 84 and 47.2% and specificity of 99 and 92.3% for EP diagnosed, respectively (32, 33). Also shown in this study, ultrasound findings of complex adnexal mass and free fluid were strong predictive factors for EP. Moreover, other findings such as endometrial thickness >14 mm (sensitivity 48%, specificity 66%) and pseudo–gestational sac and empty uterus were found to be related to EP in some studies, as well (32, 33).

To date, serum hCG became another important aiding tool for EP diagnosis and management. Many published studies focused on the concept of discriminatory zone, which was the lowest level of serum hCG that should be detected in intrauterine gestational sac by ultrasound. The absence of intrauterine gestational structure sonographic signs when serum hCG increased above the discriminatory threshold was considered diagnostic of non-viable pregnancy or EP (34). With the availability of high-resolution ultrasound, the serum hCG level of discriminatory zone has declined from 6,500 mIU/ml in transabdominal ultrasound (35) to 1,000 to 2,000 mIU/ml when approached with transvaginal ultrasound (36–38). However, Connolly (39), raised concern that currently used discriminatory serum hCG levels might be too low for use in clinical practice and may result in early offer for management that causes interrupted viable gestations. Moreover, an initial hCG level of 50% of EP in PUL was below the discrimination threshold (40). Up until recently, ideas of discriminatory zone have been varied, and single measurements were believed to limit the evaluation of PUL (41). Our study found that the proportion of patients with EP whose serum hCG <1,000 mIU/ml was less than those with non-EP (29.1 vs. 70.9%), unlike the prospective study by Malek-Mellouli et al., which found that 2.5% of EP patients have serum hCG level <1,000 mIU/ml (18). Mol et al. (42) proposed the concept of serum hCG measurement interpretation with transvaginal ultrasound findings, using hCG cutoff ≥1,500 mIU/ml in addition to positive findings in ultrasound (complex adnexal mass or fluid in cul-de-sac) (42). Surprisingly, our results found the cutoff using ≥1,000 mIU/ml as significantly relevant to EP.

Therefore, the concept of all earlier mentioned factors associated with EP proposed the idea of making diagnosis as multifactorial scores. Many published predictive models were developed. Malek-Mellouli et al. proposed the logistic regression model based on three factors including serum progesterone level, bleeding per vagina with abdominal pain, and free fluid from ultrasound finding. It resulted in sensitivity of 0.79 (95% CI = 0.62–0.91) and specificity of 0.59 (95% CI = 0.42–0.73). Unfortunately, when each one factor was compared for its prediction separately, there was no significant difference in EP prediction (18). In the latter study, Condous et al. (7, 43) developed two predictive models, which was M1 in 2004 and M4 in 2007. Analysis performed by receiver operator characteristic curve has a good prediction with sensitivity of 73.3% and specificity of 87.3% for the M1 model. Note that the positive predictive value in M1 was only 27.5%. In addition, the M4 model, using serial serum hCG at 0 and 48 h, has a better outcome in terms of sensitivity (80%) and specificity (88.6%), but there was a limitation, as longer duration of serum hCG has to be followed up. Thus, diagnosis could be delayed. Moreover, in 2010, Barnhart K. et al. (8) attempted to validate M4 model studied from the United Kingdom with a new study population in the United States. Interestingly, they found decreasing sensitivity and specificity in US population (54.8 and 87.7%) when comparing with the population in the United Kingdom, where the model was originally developed (80 and 88.6%), respectively. Gevaert et al. (44) introduced Bayesian network prediction using a more complex calculation with probabilistic model based on GA, serum hCG ratio, and serum progesterone level. However, according to its sensitivity of 77% and specificity of 83%, the model has never been tested for its validation. In our study, the predictive models including the of five parameters achieved an accuracy of 77.8%, a specificity of 91%, and sensitivity of 67%. So far, we believe that the development of a predictive model requires two important basic concepts, which are discrimination and calibration.

Our study achieves the objective of developing models for EP diagnosis among patients with symptomatic pregnancy whose first visit evaluation was PUL. Interestingly, we created the predictive scoring model that not only seem to exhibit good performance and reproducibility but was also suitable for practical use. The main goal was to provide an alternative tool for physicians dealing with PUL deciding whether intervention is necessary when EP was suspected before the rupture happens.

In a cross-sectional retrospective study, we encountered the inevitable limitation of missing values. As a result, we designed our study based on three separated domains (clinical, serum marker, and ultrasound), from which in turn we could collect and analyze each individual domain separately, thus preserving more data. In addition, the study ran over a period of 10 years; variation in defining terms for PUL could have changed overtime, as well as the treatment policy. Moreover, ultrasonographers' experiences and equipment details (environment of equipment and serum hCG interpretation equipment) may also be the causes for variation in this study. However, our present study developed in the single-center setting with adequate power for interpretation could help to minimize the confounding factor caused by differences in management of PUL patients, thus improving the consistency of the result. However, our dataset was obtained from a single medical unit, which may limit the generalization of the model. Consequently, our future work will aim not only to enhance the model internal validity with new datasets, but we also plan to generalize the model with other units. Thus, a multicenter prospective study would be planned for model validation.

## Conclusion

Our study proposed a new analytic predictive model for EP, based on a risk scoring system, revealing five potent factors that could markedly improve the basic knowledge of the disease. As far as we know, it is the first analytic model in Asian populations; regarding high accuracy and specificity, we hope this could be a tool for clinical decision making toward efficient management for EP patients. Thus, we hope that this research opens the way for diagnostic tool creation for other life-threatening diseases. Further study is required to validate the model with different patient cohorts.

## Data Availability Statement

The raw data supporting the conclusions of this article will be made available by the authors, without undue reservation.

## Ethics Statement

The studies involving human participants were reviewed and approved by Royal Thai Army Medical Department Institutional Review Board. Written informed consent for participation was not required for this study in accordance with the national legislation and the institutional requirements.

## Author Contributions

PR collected the data and performed data analysis. Both authors contributed to the conception and design of the study. Both authors interpreted data, drafted, and revised the manuscript as well as approved the version of the manuscript to be published.

## Conflict of Interest

The authors declare that the research was conducted in the absence of any commercial or financial relationships that could be construed as a potential conflict of interest.

## Abbreviations

EP: ectopic pregnancy
PUL: pregnancy of unknown location.
